# Supplementary material for: Topological Dirac nodal lines and surface charges in fcc alkaline earth metals
Source: Nat Commun. 2017 Jan 11;8:14022. doi: 10.1038/ncomms14022 (PMC5241691; doi:10.1038/ncomms14022)
Supplement: Supplementary Information — Supplementary Figures, Supplementary Notes and Supplementary References [file ncomms14022-s1.pdf]

Supplementary Note 1. Nodal lines stemming from the π Berry phase

As explained in the Methods section, nodal lines in spinless systems with inversion and time-reversal symmetries can originate from the π Berry phase. Here, we explain this mechanism. The Berry phase $\phi(\ell)$ along a loop ℓ is defined as

$$\phi(\ell) = -i \sum_{n}^{\text{occ.}} \int_{\ell} d\mathbf{k} \cdot \langle u_n(\mathbf{k}) | \nabla_{\mathbf{k}} | u_n(\mathbf{k}) \rangle \,, \tag{1}$$

where $u_n(\mathbf{k})$ is a bulk eigenstate in the n-th band, and the sum is over the occupied states. We define the Berry phase in terms of modulo 2π because it can change by an integer multiple of 2π under gauge transformation. Under a product of time-reversal and spatial inversion operations, this quantity can be transformed into $-\phi(\ell)$:

$$\phi(\ell) \equiv -\phi(\ell) \pmod{2\pi}. \tag{2}$$

This leads to quantization of the Berry phase ϕ as $\phi(\ell) \equiv 0 \text{ or } \pi \pmod{2\pi}$. Under a continuous change of ℓ in the $\mathbf{k}$ space, a jump of $\phi(\ell)$ occurs only when the band gap closes. Therefore, if the Berry phase $\phi(\ell)$ is π (mod 2π) for a certain value of ℓ, then the loop ℓ cannot continuously deform to a point without closing a gap. This condition means that closing of the gap occurs along a loop (nodal line) in $\mathbf{k}$ space, and that this loop is linked to ℓ.

We also describe in terms of an effective model a mechanism for the appearance of the nodal line. An effective model for a single valence band and a single conduction band is generally

described as follows:

$$H(\mathbf{k}) = \begin{pmatrix} a_0(\mathbf{k}) + a_z(\mathbf{k}) & a_x(\mathbf{k}) - ia_y(\mathbf{k}) \\ a_x(\mathbf{k}) + ia_y(\mathbf{k}) & a_0(\mathbf{k}) - a_z(\mathbf{k}) \end{pmatrix}$$

$$= a_0 + a_x\sigma_x + a_y\sigma_y + a_z\sigma_z. \quad (3)$$

We assume that $a_i(\mathbf{k})$ ($i = 0, x, y, z$) is a continuous function of $\mathbf{k}$. In the presence of both inversion and time-reversal symmetries, we obtain $a_y = 0$ (after an appropriate unitary transformation in some cases). Thus we have $H(\mathbf{k}) = a_0 + a_x\sigma_x + a_z\sigma_z$. The band gap closes only if the following conditions are satisfied simultaneously:

$$a_x(\mathbf{k}) = 0, \quad (4)$$

$$a_z(\mathbf{k}) = 0. \quad (5)$$

Each of these equations determines a surface in $\mathbf{k}$ space, and their intersection gives a nodal line in $\mathbf{k}$ space. In this case, a Berry phase $\phi(\ell)$ (Supplementary Eq. (1)) along a loop ℓ around the nodal line, is calculated to be equal to a change of phase of $\arg\frac{a_z}{a_x}$. This is found to be π (mod 2π), in agreement with the discussion in the previous paragraph.

Supplementary Note 2. Zak phase and polarization

2.1 Decomposition of the wavevector components with respect to the surface Brillouin zone

In preparation for the calculation in the next subsection, we show the formula for the decomposition of the wavevector $\mathbf{k}$ into the surface normal $k_\perp$ and the directions along the surface $\mathbf{k}_\parallel$.

For the calculation of the Zak phase we use the formula

$$\theta(\mathbf{k}_{\parallel}) = -i \sum_{n}^{\text{occ.}} \int_0^{b_\perp} dk_\perp \left\langle u_n(\mathbf{k}) \right| \nabla_{k_\perp} \left| u_n(\mathbf{k}) \right\rangle, \tag{6}$$

where $b_\perp$ is the width of the Brillouin zone along the direction perpendicular to the surface. However, defining the integration region when the primitive vectors are not orthogonal to each other is not straightforward. As we discussed in the main text, we consider the superstructure of the surface; furthermore, the primitive vectors may differ from the standard choice. Below, we formulate the Brillouin zone of the crystal, which takes into account the surface periodicity.

Let $\mathbf{a}_{1\parallel}$ and $\mathbf{a}_{2\parallel}$ denote the primitive vectors along the surface. If a surface superstructure is formed, then these primitive vectors should be chosen to comply with the superstructure. We then introduce another vector $\mathbf{a}'$ such that $\{\mathbf{a}_{1\parallel}, \mathbf{a}_{2\parallel}, \mathbf{a}'\}$ is a set of three-dimensional (3D) primitive vectors which takes into account the surface superstructure. Thus, it is not necessarily the primitive vectors of the 3D bulk crystal, but it is the minimal set of translation vectors which respects surface superstructure.

We then take the primitive reciprocal vectors $\{\mathbf{b}_1, \mathbf{b}_2, \mathbf{b}_\perp\}$ from $\{\mathbf{a}_{1\parallel}, \mathbf{a}_{2\parallel}, \mathbf{a}'\}$:

$$\mathbf{b}_1 = 2\pi \frac{\mathbf{a}_{2\parallel} \times \mathbf{a}'}{(\mathbf{a}_{1\parallel} \times \mathbf{a}_{2\parallel}) \cdot \mathbf{a}'}, \tag{7}$$

$$\mathbf{b}_2 = 2\pi \frac{\mathbf{a}' \times \mathbf{a}_{1\parallel}}{(\mathbf{a}_{1\parallel} \times \mathbf{a}_{2\parallel}) \cdot \mathbf{a}'}, \tag{8}$$

$$\mathbf{b}_\perp = 2\pi \frac{\mathbf{a}_{1\parallel} \times \mathbf{a}_{2\parallel}}{(\mathbf{a}_{1\parallel} \times \mathbf{a}_{2\parallel}) \cdot \mathbf{a}'} \tag{9}$$

We note that $\mathbf{b}_\perp$ is normal to the surface, whereas $\mathbf{b}_1$ and $\mathbf{b}_2$ are not necessarily along the surface

(see Supplementary Figure 1). We then project $\mathbf{b}_1$ and $\mathbf{b}_2$ onto the surface:

$$\mathbf{b}_{1\|} = \mathbf{b}_1 - \frac{\mathbf{b}_1 \cdot \mathbf{n}}{\mathbf{n} \cdot \mathbf{n}}\mathbf{n}, \tag{10}$$

$$\mathbf{b}_{2\|} = \mathbf{b}_2 - \frac{\mathbf{b}_2 \cdot \mathbf{n}}{\mathbf{n} \cdot \mathbf{n}}\mathbf{n}, \tag{11}$$

where $\mathbf{n}$ is the unit vector normal to the surface. It then follows that

$$\mathbf{a}_{i\|} \cdot \mathbf{b}_{j\|} = 2\pi\delta_{ij} \quad (i,j = 1,2); \tag{12}$$

therefore the set $\{\mathbf{b}_{1\|}, \mathbf{b}_{2\|}\}$ is a set of two-dimensional (2D) primitive reciprocal vectors for the surface, corresponding to the 2D primitive vectors along the surface, $\{\mathbf{a}_{1\|}, \mathbf{a}_{2\|}\}$. Furthermore, the 3D Brillouin zone, which is a parallelogram spanned by $\{\mathbf{b}_1, \mathbf{b}_2, \mathbf{b}_\perp\}$, is equivalent to the parallelogram spanned by $\{\mathbf{b}_{1\|}, \mathbf{b}_{2\|}, \mathbf{b}_\perp\}$, with $\mathbf{b}_\perp$ perpendicular to the surface. Therefore, we take $k_\perp$ from zero to $b_\perp$ in Supplementary Eq. (6), while $\mathbf{k}_\|$ takes a 2D wavevector within the 2D Brillouin zone spanned by $\{\mathbf{b}_{1\|}, \mathbf{b}_{2\|}\}$. We note that $b_\perp$ is equal to $2\pi/a'_\perp$, where $a'_\perp$ is a surface-normal component of $\mathbf{a}'$.

2.2 Symmetry properties of the Zak phase in three dimensions

Here we note on symmetry properties of the Zak phase. We first review the results shown in previous works [1,2], and then we discuss results for nodal-line semimetals. We first rewrite the Schrödinger equation $\mathcal{H}\psi_\mathbf{k} = E_\mathbf{k}\psi_\mathbf{k}$ in terms of the Bloch wavefunction, where $\mathcal{H}$ is the Hamiltonian, $\mathbf{k}$ is the Bloch wavevector, $\psi_\mathbf{k}$ is the wavefunction, and $E_\mathbf{k}$ is the energy. Throughout the paper, we adopt the gauge

$$\psi_{\mathbf{k}+\mathbf{G}} = \psi_\mathbf{k}, \tag{13}$$

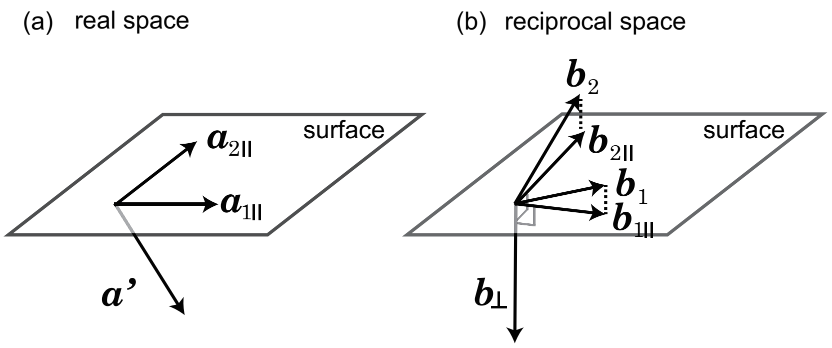

Supplementary Figure 1: **Primitive vectors.** Primitive vectors in **a**, **b**, real space and reciprocal space, respectively, used in our calculation

where $\mathbf{G}$ is any reciprocal lattice vector [1,2]; this choice of gauge is necessary for relating the Zak phase $\theta(\mathbf{k}_{||})$ to polarization. We then obtain

$$\hat{H}_{\mathbf{k}} u_{\mathbf{k}} = E_{\mathbf{k}} u_{\mathbf{k}}, \tag{14}$$

where $\psi_{\mathbf{k}} = u_{\mathbf{k}} e^{i\mathbf{k}\cdot\mathbf{r}}$ and $\hat{H}_{\mathbf{k}} \equiv e^{-i\mathbf{k}\cdot\mathbf{r}} \mathcal{H} e^{i\mathbf{k}\cdot\mathbf{r}}$. The choice of gauge in Supplementary Eq. (13) is rewritten as $u_{\mathbf{k}} = u_{\mathbf{k}+\mathbf{G}} e^{i\mathbf{k}\cdot\mathbf{r}}$.

When the inversion and time-reversal symmetries are present in spinless systems, the Zak phase $\theta(C)$ around any closed loop C is quantized as follows:

$$\theta(C) \equiv -i \sum_{n}^{\text{occ.}} \oint_C d\mathbf{k} \cdot \langle u_n(\mathbf{k}) | \nabla_{\mathbf{k}} | u_n(\mathbf{k}) \rangle = n\pi \ \ (n: \text{ integer}). \tag{15}$$

In particular, the Berry phase around the nodal line (contour c in Supplementary Figure 2) is π; because of the above quantization, the nodal line is topologically protected. Thus the Zak phases (Berry phase) along the surface normal $k_\perp$ changes by π, when $\mathbf{k}_{||}$ is changed across the projection

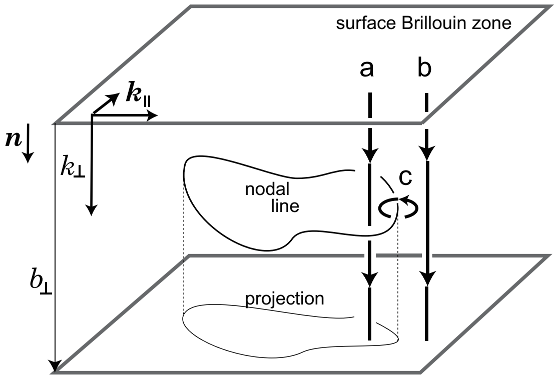

Supplementary Figure 2: **Zak and Berry phases.** Relation between the Zak phases (Berry phase) along the surface normal (paths a, b) and the Berry phase around the nodal line.

of the nodal line onto the surface. In Supplementary Figure 2 the Zak phases for the paths a and b differ by π, which is the Berry phase around the loop c around the nodal line.

In the following we show the effects of the symmetries of the system on $\theta(\mathbf{k}_{\|})$

(i) Inversion symmetry

When the system has inversion symmetry, we have

$$[\mathcal{P}, \mathcal{H}] = 0 \;\Rightarrow\; \mathcal{P}\hat{H}_{\mathbf{k}}\mathcal{P}^{-1} = \hat{H}_{-\mathbf{k}}, \tag{16}$$

where $\mathcal{P}$ is the inversion operator. We can then derive the relationship for the Zak phase:

$$\theta(\mathbf{k}_{\|}) \equiv -\theta(-\mathbf{k}_{\|}) \pmod{2\pi}. \tag{17}$$

Compared with the present result, the results in a previous work [3] contains an additional term. This is due to the choices of gauge; the above study [3] adopted the gauge $u_{\mathbf{k}} = u_{\mathbf{k}+\mathbf{G}}$, whereas we adopt

the gauge in Supplementary Eq. (13), which is directly related to the polarization.

(ii) Time-reversal symmetry

When the system has time-reversal symmetry, we have

$$[\mathcal{K}, \mathcal{H}] = 0 \ \Rightarrow \ \mathcal{K}\hat{H}_{\mathbf{k}}\mathcal{K} = \hat{H}_{-\mathbf{k}}, \tag{18}$$

where $\mathcal{K}$ is the complex conjugation. Here we focus on spinless systems, for which the time-reversal operation is represented as $\mathcal{K}$. The Zak phase then satisfies

$$\theta(-\mathbf{k}_{\|}) \equiv \theta(\mathbf{k}_{\|}) \pmod{2\pi}. \tag{19}$$

This is the same as that in the above study [3], although the gauges are different from ours.

(iii) Inversion and time-reversal symmetries

When the system has both time-reversal and inversion symmetries, Eqs. (17) and (19) yield from the results in (i) and (ii)

$$\theta(\mathbf{k}_{\|}) \equiv 0 \text{ or } \pi \pmod{2\pi}. \tag{20}$$

We consider an implication of Supplementary Eq. (20) for insulators and for nodal-line semimetals in the following discussion.

In a previous work [2], the relationship between the Zak phase and the surface polarization charge density σ was found. The surface polarization charge density, i.e. the surface normal

component of the polarization vector, is given by

$$\sigma = \sigma_{\text{ion}} + \sigma_e \tag{21}$$

where σ_{ion} is an ionic contribution from surface atoms, and σ_e represents an electronic contribution

$$\sigma_e = \int \frac{d^2 k_\parallel}{(2\pi)^2} \sigma_e(\mathbf{k}_\parallel), \ \ \sigma_e(\mathbf{k}_\parallel) \equiv \frac{-e}{2\pi}\theta(\mathbf{k}_\parallel) \ \ (\text{mod } e). \tag{22}$$

If we regard the system at fixed $\mathbf{k}_\parallel$ to be a one-dimensional system, $\sigma_e(\mathbf{k}_\parallel)$ is an electronic surface charge density for the one-dimensional subssystem at $\mathbf{k}_\parallel$ [2].

We first consider insulators, assuming that there is no surface state that crosses the Fermi energy. Thus, $\sigma_e(\mathbf{k}_\parallel)$ does not have a jump as a function of $\mathbf{k}_\parallel$. According to Supplementary Eq. (20), $\sigma_e(\mathbf{k}_\parallel)$ is therefore independent of $\mathbf{k}_\parallel$:

$$\sigma_e(\mathbf{k}_\parallel) = N\frac{e}{2}, \tag{23}$$

where N is an integer constant. Hence, the surface charge density is $\sigma_e = \frac{Ne}{2A_{\text{surface}}}$ where A_{surface} is an area of the surface unit cell [2]. Although N can be any integer, it is physically expected to vanish in almost all insulators, because nonzero N corresponds to a large polarization, which leads to chemical or electronic instability. Thus $N = 0$ is expected of stable electronic states; so far, no insulator is known to have nonzero integer N, which means a huge surface polarization.

In materials with nodal lines which are the focus of the present work, the Zak phase jumps by π at the nodal lines; therefore, there is always a region with $\theta(\mathbf{k}_\parallel) \equiv 0$ (mod 2π) and one

with $\theta(\mathbf{k}_{\|}) \equiv \pi$ (mod 2π). The latter region leads to an appreciable polarization. In nodal-line semimetals, the bulk electronic carriers and ions eventually screen the polarization, but large deformation of the lattice structure and surface dipoles occur. We expect this to lead to large Rashba splitting if adatoms with large spin-orbit coupling are present, as indicated in the main text.

Supplementary Note 3. Surface termination and choice of the unit cell

The surface polarization charge density σ_e is related to the polarization vector $\mathbf{P}$ by $\sigma = P_{\perp} \equiv \mathbf{P} \cdot \mathbf{n}$, where $\mathbf{n}$ is a unit vector normal to the surface. Even when the direction of the surface plane is fixed, such as in (111) or (001), there are possibilities for surface terminations. Moreover, there are various possible choices for the unit cell for a given surface termination.

The dependence on the choice of unit cell is discussed in another study [2]. In summary, results of this work [2] indicate that the polarization $\sigma(= P_{\perp})$ at a fixed surface termination is independent of the choice of the unit cell of the bulk. That is, whereas σ is independent of the choice of the unit cell at a fixed surface termination, the contributions of σ_e and σ_{ion} in Supplementary Eq. (21) may depend on the unit cell choice.

On the other hand, σ generally changes with the surface termination. In the following discussion, we consider several cases of surface terminations for the (001) and (111) surfaces. For the calculations we always choose the unit cell in such a way that there are no additional ‘surface

atoms', which are excess atoms that are not covered by translations of the unit cell [2]. With such a choice of unit cell, we always have $\sigma_{\rm ion} = 0$ and we only have to consider the dependence of the electronic part σ_e.

Thus, the unit cell is chosen accordingly in the following discussion of various surface terminations. Because the choice of unit cell corresponds to the unitary transformation of the Hamiltonian, it affects the Zak phase in the following manner [3]. Suppose the unit structure consists of N atoms at $\{\mathbf{r}_1, \cdots, \mathbf{r}_N\}$. If the unit cell convention is changed to $\{\mathbf{r}_1 + \epsilon_1, \cdots, \mathbf{r}_N + \epsilon_N\}$ where ϵ_a ($a = 1, \cdots, N$) are translation vectors of the crystal, then the change in the Zak phase $\Delta\theta'(\mathbf{k}_\parallel)$ is expressed as follows [3]:

$$\Delta\theta'(\mathbf{k}_\parallel) = -2\pi \sum_{a=1}^{N} \epsilon_a^\perp \rho_a(\mathbf{k}_\parallel), \tag{24}$$

where $\epsilon_a^\perp$ is a surface-normal component of ϵ_a, and

$$\rho_a(\mathbf{k}_\parallel) \equiv \sum_{m}^{\rm occ.} \int_0^{b_\perp} \frac{dk_\perp}{2\pi} \langle u_{n\mathbf{k}} | \mathcal{P}_a | u_{n\mathbf{k}} \rangle. \tag{25}$$

Here $\mathcal{P}_a$ ($a = 1, \cdots, N$) is a projection operator projecting onto the atom a.

In the present framework, ϵ_a is a translation vector, which is a linear combination of $\{\mathbf{a}_{1\parallel}, \mathbf{a}_{2\parallel}, \mathbf{a}'\}$ with integer coefficients. Among these primitive vectors, only $\mathbf{a}'$ has a nonzero surface-normal component. Hence, $\epsilon_a^\perp$ is an integer multiple of $a'_\perp$.

3.1 (001) surface

On the (001) surface, a $\sqrt{2} \times \sqrt{2}$ structure is formed when half of the surface atoms are

depleted. Therefore, we consider from the outset the unit cell for the 2D surface with an enlarged unit cell for the $\sqrt{2}\times\sqrt{2}$ structure. Let a denote the lattice constant for the cubic unit cell of the fcc lattice. We take one of the surface atoms to be an origin, and the surface to be along the xy plane. The primitive vectors for the 2D surface can then be taken as:

$$\mathbf{a}_{1\|} = a(1,0,0),\ \mathbf{a}_{2\|} = a(0,1,0) \tag{26}$$

The other primitive vector is then given by $\mathbf{a}' = a(\frac{1}{2},0,\frac{1}{2})$. The unit cell spanned by $\{\mathbf{a}_{1\|},\mathbf{a}_{2\|},\mathbf{a}'\}$ contains two atoms. Thus, we have

$$\mathbf{b}_1 = \frac{2\pi}{a}(1,0,-1),\ \ \mathbf{b}_2 = \frac{2\pi}{a}(0,1,0),\ \ \mathbf{b}_\perp = \frac{4\pi}{a}(0,0,1). \tag{27}$$

Additionally,

$$\mathbf{b}_{1\|} = \frac{2\pi}{a}(1,0,0),\ \ \mathbf{b}_{2\|} = \frac{2\pi}{a}(0,1,0). \tag{28}$$

For the perfect (001) surface on the xy plane (Supplementary Figure 3a), the unit structure consists of the two atoms at $(0,0,0)$ and $\mathbf{c} = a(-\frac{1}{2},\frac{1}{2},0)$. Let us denote the two sublattices I and II, which belong to the points 0 and $\mathbf{c}$, respectively. When the surface atoms at $a(m+\frac{1}{2},n+\frac{1}{2})$ (m,n: integer) become depleted (Supplementary Figure 3b), the surface forms a $\sqrt{2}\times\sqrt{2}$ structure, and the unit structure consists of the two atoms at $(0,0,0)$ and $\mathbf{c}+\mathbf{a}' = a(0,\frac{1}{2},\frac{1}{2})$. Both choices of the unit structure are inversion-symmetric; therefore, the Zak phase in both cases is quantized as 0 or π (mod 2π). Thus, the atom in sublattice II in the unit structure in Supplementary Figure 3b is shifted from those in Supplementary Figure 3a by $\mathbf{a}'$, and

$$\Delta\theta'(\mathbf{k}_\|) = -2\pi a'_\perp \rho_{\rm II}(\mathbf{k}_\|), \tag{29}$$

$$\rho_{\rm II}(\mathbf{k}_{\|}) \equiv \sum_{m}^{\rm occ.} \int_0^{b_\perp} \frac{dk_\perp}{2\pi} \langle u_{n\mathbf{k}}|\mathcal{P}_{\rm II}|u_{n\mathbf{k}}\rangle. \tag{30}$$

Noting that the two sublattices are equivalent, we obtain $\langle u_{n\mathbf{k}}|\mathcal{P}_{\rm a}|u_{n\mathbf{k}}\rangle = \frac{1}{2}\langle u_{n\mathbf{k}}|u_{n\mathbf{k}}\rangle = \frac{1}{2}$ ($a = \rm I, II$) and $\rho_{\rm II}(\mathbf{k}_{\|}) = \sum_m^{\rm occ.} \frac{1}{2}\frac{b_\perp}{2\pi} = \frac{N_{\rm occ.}}{2}\frac{b_\perp}{2\pi}$, where $N_{\rm occ.}$ is the number of occupied bands. Thus,

$$\Delta\theta(\mathbf{k}_{\|}) = -\frac{N_{\rm occ.}}{2} a'_\perp b_\perp = -\frac{N_{\rm occ.}}{2} 2\pi \tag{31}$$

Lastly we note that the unit cell is doubled from the original fcc unit cell; therefore, $N_{\rm occ.}$ is even. Thus the Zak phase is unchanged, i.e. $\Delta\theta(\mathbf{k}_{\|}) \equiv 0 \pmod{2\pi}$, in accordance with the *ab initio* calculation in the main text. This invariance of the Zak phase is natural because it is a bulk quantity independent of the surface.

3.2 (111) surface

When one-third or two-thirds of the surface atoms on the (111) surface are depleted, a $\sqrt{3} \times \sqrt{3}$ structure is formed. Therefore, we consider from the outset the unit cell for the 2D surface with an enlarged unit cell for the $\sqrt{3} \times \sqrt{3}$ structure. We take one of the surface atoms to be an origin, and the surface to be along the xy plane. While the standard choice for the primitive vectors are $\tilde{\mathbf{a}}_{1\|} = \frac{a}{\sqrt{2}}(0,1,0),\ \tilde{\mathbf{a}}_{2\|} = \frac{a}{2\sqrt{2}}(-\sqrt{3},1,0)$, the primitive vectors for the $\sqrt{3} \times \sqrt{3}$ structure can be

$$\mathbf{a}_{1\|} = \frac{a}{2\sqrt{2}}(\sqrt{3},3,0),\ \mathbf{a}_{2\|} = \frac{a}{2\sqrt{2}}(-\sqrt{3},3,0) \tag{32}$$

The other primitive vector is then given by $\mathbf{a}' = \frac{a}{6\sqrt{2}}(\sqrt{3},3,2\sqrt{6})$. The unit cell spanned by $\{\mathbf{a}_{1\|}, \mathbf{a}_{2\|}, \mathbf{a}'\}$ contains three atoms. Thus, we have

$$\mathbf{b}_1 = \frac{2\pi}{3a}(\sqrt{6},\sqrt{2},-\sqrt{3}),\ \ \mathbf{b}_2 = \frac{2\sqrt{2}\pi}{3a}(-\sqrt{3},1,0), \tag{33}$$

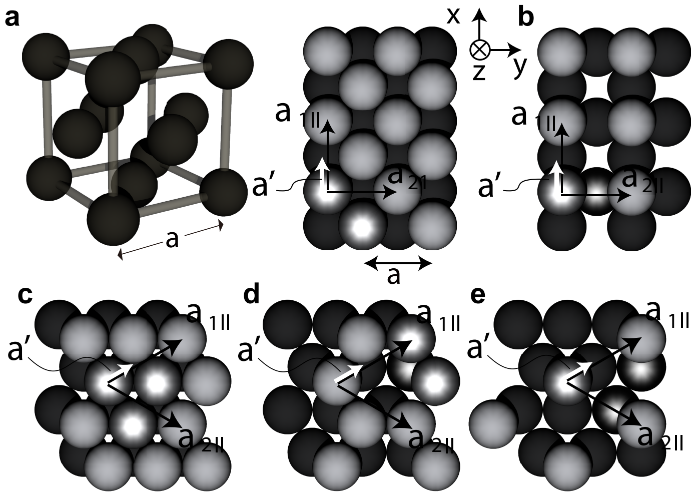

Supplementary Figure 3: **Surface structure and primitive vectors.** **a**, Crystal structure of fcc Ca, Sr and Yb, and that of the (001) surface (black circles) with surface atoms (grey circles). **b**, The same surface orientation but with one-half of the atoms per unit cell on the surface. **c**, Crystal structure of the (111) surface (black circles) with surface atoms (grey circles). **d**, **e**, The same surface orientation but with one-third and two-thirds of the atoms, respectively, per unit cell on the surface. In **a**-**e**, the primitive vectors $\mathbf{a}_{1\|}$, $\mathbf{a}_{2\|}$ along the surface are shown as black arrows, while the other primitive vector $\mathbf{a}'$ is shown as a white arrow. Here, the choice of the unit structure is marked by the circles with gradation, i.e. in **a** and **b** the unit structure consists of two atoms, while in **c**-**e** it consists of three atoms.

$$\mathbf{b}_{\perp} = \frac{2\sqrt{3}\pi}{a}(0,0,1) \tag{34}$$

and

$$\mathbf{b}_{1\|} = \frac{2\sqrt{2}\pi}{3a}(\sqrt{3},1,0), \;\; \mathbf{b}_{2\|} = \frac{2\sqrt{2}\pi}{3a}(-\sqrt{3},1,0). \tag{35}$$

For the perfect (111) surface on the xy plane (Supplementary Figure 3c), the unit structure consists of the three atoms at $\{0, \tilde{\mathbf{a}}_{1\|}, \tilde{\mathbf{a}}_{2\|}\}$. Let us denote the three sublattices I, II and III which belongs to the points 0, $\tilde{\mathbf{a}}_{1\|}$ and $\tilde{\mathbf{a}}_{2\|}$, respectively When the one-third of the atoms become depleted (Supplementary Figure 3d), the unit structure consists of the three atoms at $\{0, \tilde{\mathbf{a}}_{1\|} + \mathbf{a}', \tilde{\mathbf{a}}_{2\|}\}$. Thus, the atom in sublattice II is shifted by $\mathbf{a}'$ in the new selected of unit structure, and

$$\Delta\theta(\mathbf{k}_{\|}) = -2\pi a'_{\perp}\rho_{\mathrm{II}}(\mathbf{k}_{\|}), \tag{36}$$

$$\rho_{\mathrm{II}}(\mathbf{k}_{\|}) \equiv \sum_{m}^{\mathrm{occ.}} \int_0^{b_\perp} \frac{dk_\perp}{2\pi} \langle u_{n\mathbf{k}}|\mathcal{P}_{\mathrm{II}}|u_{n\mathbf{k}}\rangle. \tag{37}$$

Noting that the three sublattices are equivalent, we obtain $\langle u_{n\mathbf{k}}|\mathcal{P}_{\mathrm{a}}|u_{n\mathbf{k}}\rangle = \frac{1}{3}\langle u_{n\mathbf{k}}|u_{n\mathbf{k}}\rangle = \frac{1}{3}$ ($a = \mathrm{I}, \mathrm{II}, \mathrm{III}$), and $\rho_{\mathrm{II}}(\mathbf{k}_{\|}) = \sum_m^{\mathrm{occ.}} \frac{1}{3}\frac{b_\perp}{2\pi} = \frac{N_{\mathrm{occ.}}}{3}\frac{b_\perp}{2\pi}$, where $N_{\mathrm{occ.}}$ is the number of occupied bands. Thus,

$$\Delta\theta(\mathbf{k}_{\|}) = -\frac{N_{\mathrm{occ.}}}{3} a'_{\perp} b_{\perp} = -\frac{N_{\mathrm{occ.}}}{3} 2\pi \tag{38}$$

Lastly we note that the unit cell is tripled from the original fcc unit cell; therefore, $N_{\mathrm{occ.}}$ is a integer multiple of three. Consequently, the Zak phase is unchanged: $\Delta\theta(\mathbf{k}_{\|}) \equiv 0 \pmod{2\pi}$. This invariance of the Zak phase is natural because it is a bulk quantity independent of the surface.

When the two-thirds of the atoms are depleted (Supplementary Figure 3e), the unit structure

consists of the three atoms at $\{0, \tilde{\mathbf{a}}_{1\parallel} + \mathbf{a}', \tilde{\mathbf{a}}_{2\parallel} + \mathbf{a}'\}$. Using a similar calculation we obtain

$$\Delta\theta'(\mathbf{k}_{\parallel}) = -2\pi(a'_{\perp}\rho_{\mathrm{II}}(\mathbf{k}_{\parallel}) + a'_{\perp}\rho_{\mathrm{III}}(\mathbf{k}_{\parallel})), \tag{39}$$

$$\rho_{\mathrm{II}}(\mathbf{k}_{\parallel}) = \rho_{\mathrm{III}}(\mathbf{k}_{\parallel}) = \frac{N_{\mathrm{occ.}}}{3}\frac{b_{\perp}}{2\pi}. \tag{40}$$

Therefore,

$$\Delta\theta(\mathbf{k}_{\parallel}) = -\frac{2N_{\mathrm{occ.}}}{3}2\pi \equiv 0 \pmod{2\pi} \tag{41}$$

and the Zak phase is unchanged, in accordance with the *ab initio* calculation in the main text. We also note that in these three choices of the unit structure, the Zak phase is quantized as 0 or π (mod 2π).

Supplementary Note 4. $\mathbb{Z}_2$ topology of nodal lines in alkaline-earth metals

In a previous work [4], a $\mathbb{Z}_2$ topological number is defined for each nodal line in spinless systems with both inversion and time-reversal symmetries. If it is nontrivial, then the nodal line cannot vanish by itself after shrinking to a point. This $\mathbb{Z}_2$ topological number can be defined for each nodal line in Ca, when the spin-orbit interaction is neglected. This has been found to be trivial; the nodal lines around the L points disappear upon addition of an artificial potential for the $4s$ orbital (see Supplementary Figures 4a-c).

Similar analysis of the nodal lines around 5 eV in Ag (Supplementary Figure 4d) shows that it is also $\mathbb{Z}_2$-trivial as defined by the above study [4]. Supplementary Figures 4e and f show the disappearance of the nodal line with the decrease in the on-site potential of the $5s$ orbital.

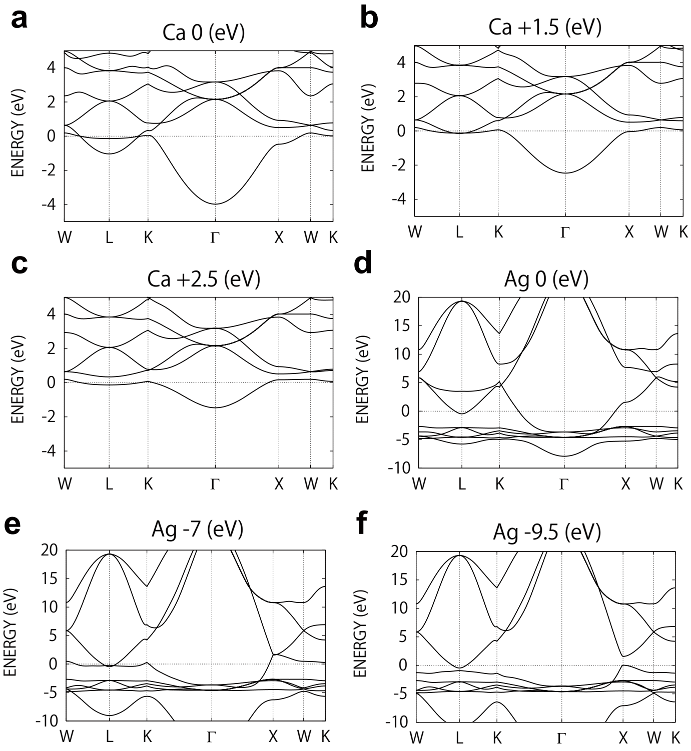

Supplementary Figure 4: **Disappearance of nodal lines by adding the on-site potential.** **a**, Electronic band structure of Ca in the LDA. **b**, **c**, Electronic band structure of Ca, depicting addition of 1.5 and 2.5 eV, respectively, to the on-site potential of the s orbital. **d**, Electronic band structure of Ag in the LDA. **e**, **f**, Electronic band structure of Ag, depicting subtraction of 7 and 9.5 eV, respectively, from the on-site potential of the s orbital.

Supplementary Note 5. Screening in nodal-line semimetals

We have shown in the main text that when the nodal-line semimetal is regarded as a set of independent one-dimensional systems for individual values of $\mathbf{k}_{\|}$, within a $\mathbf{k}$-space region of π Zak phase, there is an appreciable polarization of $\pm e/2$. Nevertheless, the polarization charges at the surface are eventually screened since the entire system is a semimetal with carriers. In this section we consider screening of the surface polarization charges by carriers in nodal-line semimetals. For simplicity, we consider the nodal-line semimetal with its nodal line being a circle in the k_x-k_y plane with radius k_0, assuming the dispersion perpendicular to the nodal line to be linear with velocity v_0. The dispersion can then be represented as $E = \pm\hbar v_0\sqrt{(\sqrt{k_x^2+k_y^2}-k_0)^2+k_z^2}$. Thus, the density of states is $\nu(E) = C|E|$, with $C = \frac{k_0}{2\pi v_0^2\hbar^2}$ per unit volume. In calcium there are four nodal lines; therefore, the constant C is multiplied by the number of nodal lines $g(=4)$.

The Poisson equation is

$$\frac{d^2V}{dz^2} = \frac{e}{\varepsilon_0\varepsilon}\rho \tag{42}$$

where $\rho(z)$ is the charge density, and V is the potential energy for electrons [5]. We set the z-axis normal to the surface of the semimetal, with $z = 0$ representing the surface.

We suppose that the polarization charge appears at the surface because of the presence of nodal lines, with polarization charge density $\sigma_{\rm s}$. As we have shown in the main text, for example, the nodal line depletes electrons on the Ca surface within the area in $\mathbf{k}$ space surrounded by the nodal lines (shown as the shaded region in Supplementary Figure 3h), and the polarization charge

is positive, that is, $\sigma_s > 0$. Electron carriers are then induced near the surface because of this positive surface charge, and $V(z) < 0$ is expected for the region near the surface. The following equation relates the charge density ρ to the potential V,

$$\rho(z) = -en(z),\ n(z) = \int_0^\infty f_{\mathrm{F}}(E,z)\nu(E)dE, \tag{43}$$

where $f_{\mathrm{F}}(E,z) = \frac{1}{e^{\beta(E-E_F+V(z))}+1}$ is the Fermi distribution function in the presence of potential $V(z)$. For simplicity we consider zero temperature and $E_F = 0$ (i.e. at the nodal line). We thus have

$$n(z) = \frac{1}{2}CV(z)^2 \tag{44}$$

From Eqs. (42) (43) and (44), we obtain

$$\frac{d^2V}{dz^2} = -\frac{e^2C}{2\varepsilon_0\varepsilon}V^2 \tag{45}$$

with boundary conditions $V(z=\infty) = 0$, $V'(z=0) = \frac{e}{\varepsilon_0\varepsilon}\sigma_{\mathrm{s}}$. The solution is

$$V(z) = -\frac{e\lambda\sigma_{\mathrm{s}}}{2\varepsilon_0\varepsilon}\frac{1}{(1+z/\lambda)^2}, \tag{46}$$

where $\lambda = \left(\frac{24\varepsilon_0^2\varepsilon^2}{e^3C\sigma_{\mathrm{s}}}\right)^{1/3}$ represents a screening length. The charge distribution is

$$n(z) = \frac{1}{2}CV^2 = \frac{C}{8}\left(\frac{e\lambda\sigma_{\mathrm{s}}}{\varepsilon_0\varepsilon}\right)^2\frac{1}{(1+z/\lambda)^4} \tag{47}$$

The spatial dependence of the potential $V(z)$ and electron density $n(z)$ are plotted in Supplementary Figure 5. The total induced charge density is calculated as $\sigma_{\mathrm{ind}} = -e\int_0^\infty ndz = -\sigma_s$. Therefore, the induced electronic distribution totally screens the positive polarization charge at the

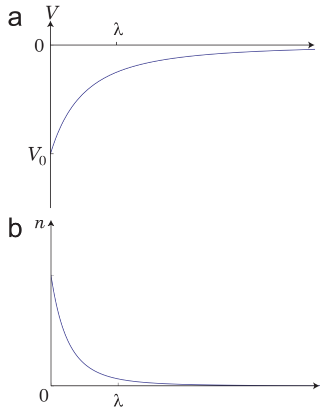

Supplementary Figure 5: **Electronic potential and density due to screening of surface charges.** **a**, **b**, Spatial dependence of the potential $V(z)$ and electron density, respectively.

surface. Meanwhile, there remains a finite dipole moment, the density of which is calculated as follows:

$$-e\int_0^\infty nzdz = -\frac{\sigma_{\rm s}\lambda}{2} \tag{48}$$

Upon setting $\varepsilon = 5$, $\sigma_{\rm s} \sim 0.243e/A_{\rm surface}$, $A_{\rm surface} = 1.5 \times 10^{-19}{\rm m}^2$, $v_0 \sim 3 \times 10^5{\rm m\ s}^{-1}$, and $k_0 \sim 0.24{\rm nm}^{-1}$ for rough estimates for calcium at 7.5 GPa, the screening length is estimated as $\lambda \sim 0.24{\rm nm}$, i.e. on the order of a lattice constant. The depth of the potential $V_0 \equiv V(z=0) = -\frac{e\lambda\sigma_{\rm s}}{2\varepsilon_0\varepsilon}$ is approximately -0.77 eV. The dipole density per surface unit cell is $0.243e \cdot 0.24{\rm nm}/2 = 4.7 \times 10^{-21}C \cdot {\rm nm}$, and the electric field at the surface is $-2V_0/(e\lambda) = 6.4{\rm V\ nm}^{-1}$.

Thus far, we have studied screening by bulk carriers. We found that the dipoles are formed at the surface, and that the electronic potential is lower near the surface as shown in Supplementary Figure 5, This property affects surface states, if any, as discussed below. Supplementary Figure 6 shows results from full self-consistent slab calculations with the lattice being fixed for the band

a **b**

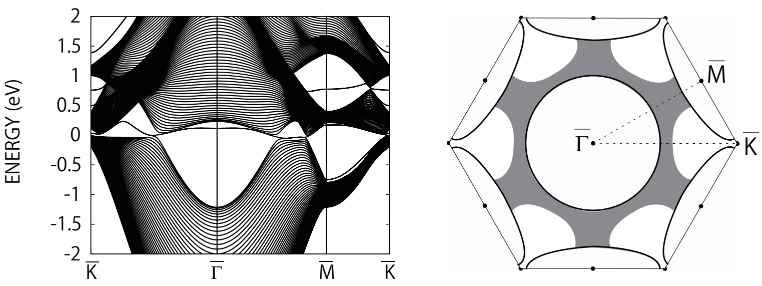

Supplementary Figure 6: **Electronic relaxation on the surface of Ca. a**, Electronic band structure of Ca at 7.5 GPa for the (111) surface in the LDA, with the lattice is fixed. **b**, The region in the surface Brillouin zone (grey); where the surface states descend below the Fermi energy by relaxation. The solid curves are projections of nodal lines.

structure of the Ca slab at 7.5GPa with (111) surfaces. Comparing Supplementary Figure 3**g** (without electronic relaxation) and Supplementary Figure 6**a** (with electronic relaxation), we see that surface states descend to the Fermi energy, which is within the grey region inSupplementary Figure 6**b**, and that some of the surface states descend even below the Fermi energy, becoming occupied (shown as the shaded region in Supplementary Figure 6**b**). This lowering of surface states is attributed to the negative potential $V(z)$ near the surface. Because the potential $V(z)$ is close to the surface, the surface states with shorter penetration depth are more affected by the potential $V(z)$. The maximum of the energy shift of the surface state is expected to be V_0; it has been estimated to be around -0.77eV. This estimate is in good agreement with the energy shift of the surface states between Supplementary Figure 3**g** (without electronic relaxation) and Supplementary Figure 6**a** (with electronic relaxation). To summarize, the bulk carriers partially

screen the surface polarization charge due to the nodal lines, leaving behind dipoles at the surface. This induces an electronic potential which affects surface states, if there are any surface states within the energy scale of the potential $V(z)$ at the surface.

Supplementary References

1. Zak, J. Berry's phase for Energy Bands in Solids. *Phys. Rev. Lett.* **62**, 2747–2750 (1982).

2. Vanderbilt, D. & King-Smith, R. D. Electric polarization as a bulk quantity and its relation to surface charge. *Phys. Rev. B* **48**, 4442–4455 (1993).

3. Kariyado, T. & Hatsugai, Y. Symmetry-protected quantization and bulk-edge correspondence of massless Dirac fermions: Application to the fermionic Shastry-Sutherland model. *Phys. Rev. B* **88**, 245126 (2013).

4. Fang, C., Chen, Y., Kee, H.-Y. & Fu, L. Topological nodal line semimetals with and without spin-orbital coupling. *Phys. Rev. B* **92**, 081201 (2015).

5. Zöllner, J. -P., Übensee, H., Paasch, G., Fiedler, T. & Gobsch, G. A Novel Self-Consistent Theory of the Electronic Structure of Inversion Layers in InSb MIS Structures, *Phys. Stat. Sol. (b)* **134**, 837–845 (1986).
